# Real-World Pharmacological Patterns and Residual Cardiometabolic Risk in Patients with Type 2 Diabetes in Romania

**DOI:** 10.3390/diseases14100387

**Published:** 2026-10-21

**Authors:** Bianca-Lăcrimioara Petca, Paula-Alexandra Popovici, Andreea Diana Igna, Timea Claudia Ghitea, Mihaela Simona Popoviciu

**Affiliations:** 1 Doctoral School of Biological and Biomedical Sciences, University of Oradea, 1 University Street, 410087 Oradea, Romania; criste.biancalacrimioara@student.uoradea.ro (B.-L.P.); cipleu.paulaalexandra@student.uoradea.ro (P.-A.P.); igna.andreeadiana@student.uoradea.ro (A.D.I.); 2 Pharmacy Department, Faculty of Medicine and Pharmacy, University of Oradea, 1 University Street, 410087 Oradea, Romania; 3 Department of Preclinical Disciplines, Faculty of Medicine and Pharmacy, University of Oradea, 410028 Oradea, Romania; mihaela.popoviciu@didactic.uoradea.ro

**Keywords:** type 2 diabetes mellitus, residual cardiovascular risk, residual renal risk, SGLT2 inhibitors, GLP-1 receptor agonists, pharmacological treatment patterns, statin therapy, real-world evidence, therapeutic gaps, cardio-renal–metabolic syndrome

## Abstract

Background: Contemporary management of type 2 diabetes mellitus (T2DM) has shifted from a glucose-centered approach toward integrated cardio-renal–metabolic protection. Although sodium–glucose cotransporter-2 (SGLT2) inhibitors, glucagon-like peptide-1 receptor agonists (GLP-1 RAs), and intensive lipid-lowering therapies have demonstrated substantial cardiovascular and renal benefits, implementation in routine outpatient care and the burden of persistent residual risk remain insufficiently characterized in Romania. Objectives: We aimed to evaluate routine-care pharmacological treatment patterns, identify residual cardio-renal–metabolic risk despite ongoing therapy, determine independent correlates of persistent adverse outcomes, and explore potential therapeutic gaps in adults with T2DM. Methods: This cross-sectional observational study included 232 unique adults with T2DM attending a specialized outpatient diabetes clinic in Romania between 1 October 2024 and 19 February 2026. One eligible outpatient record per participant was analyzed; when multiple records were available, the earliest eligible record in the exported dataset was used as the index visit. No a priori sample-size calculation was performed. Pharmacological therapies, cardiovascular and renal phenotypes, metabolic biomarkers, and clinical outcomes were extracted from routinely collected electronic medical records. Residual risk was evaluated using glycemic, lipid, renal, obesity, and blood-pressure targets. Multivariable logistic regression analyses identified independent correlates of persistent cardio-metabolic abnormalities. An exploratory cumulative residual-risk count was constructed by summing seven clinically relevant uncontrolled risk factors. Results: Biguanides were the most frequently prescribed glucose-lowering agents (53.0%), followed by SGLT2 inhibitors (44.8%), insulin (32.3% of the total cohort), GLP-1 receptor agonists (15.5%), and DPP-4 inhibitors (14.7%). Statins were prescribed to 48.7% of participants. SGLT2 inhibitor use did not differ significantly according to established atherosclerotic cardiovascular disease, heart failure, or chronic kidney disease phenotypes, whereas GLP-1 receptor agonists were prescribed more frequently in participants with obesity (22.0% vs. 7.6%, *p* = 0.003). Clinically important residual risk persisted across all treatment groups. Among SGLT2 inhibitor users, 57.3% had HbA1c ≥ 7%, while 27.9% remained albuminuric. Among insulin-treated participants, 84.0% had HbA1c ≥ 7%; reduced eGFR and albuminuria were also more frequent than among non-users. Despite statin therapy, 33.6% of treated participants continued to exhibit LDL cholesterol ≥ 100 mg/dL. In multivariable logistic regression, insulin therapy was associated with higher odds of HbA1c ≥ 7% (adjusted odds ratio [aOR] 7.41, 95% confidence interval [CI] 3.50–15.65), while SGLT2 inhibitor therapy was associated with lower odds of albuminuria (aOR 0.54, 95% CI 0.30–0.95). Absence of statin-containing therapy was associated with higher odds of LDL cholesterol ≥ 100 mg/dL (aOR 6.01, 95% CI 3.38–10.66). Absence of statin-containing therapy was associated with higher odds of residual LDL cholesterol ≥ 100 mg/dL (aOR 4.21, 95% CI 2.34–7.58). Seventy-two percent of participants presented at least three persistent cardio-renal–metabolic abnormalities, while 24.2% exhibited five or more. More than half of participants with established atherosclerotic cardiovascular disease, heart failure, or chronic kidney disease were not receiving either an SGLT2 inhibitor or a GLP-1 receptor agonist. Conclusions: Significant residual cardio-renal–metabolic risk persists in adults with T2DM despite use of contemporary pharmacological therapies. Disease severity markers and treatment exposure were associated with persistent adverse outcomes, but the cross-sectional design does not establish causality. The study identified potentially modifiable therapeutic gaps among patients with established cardiovascular and renal disease. Comprehensive and repeatedly reassessed cardio-renal–metabolic management, integrating pharmacological optimization with individualized risk stratification, may improve implementation of guideline-directed therapy and reduce long-term complications in routine clinical practice.

## 1. Introduction

Type 2 diabetes mellitus (T2DM) has evolved from a disease primarily characterized by hyperglycemia into a complex cardio-renal–metabolic syndrome associated with substantial cardiovascular morbidity, chronic kidney disease, heart failure, and premature mortality. Although remarkable advances in glucose-lowering therapies have transformed diabetes management over the past decade, patients continue to experience considerable residual cardiovascular and renal risk despite achieving recommended glycemic targets. This observation has shifted the therapeutic focus from glucose-centered care toward comprehensive management of interconnected metabolic, cardiovascular, and renal abnormalities [1,2,3].

Large cardiovascular outcome trials have demonstrated that sodium–glucose cotransporter-2 (SGLT2) inhibitors and glucagon-like peptide-1 receptor agonists (GLP-1 RAs) provide clinically meaningful reductions in cardiovascular events, heart failure hospitalization, progression of chronic kidney disease, and all-cause mortality, extending their benefits beyond glucose lowering alone. Consequently, contemporary recommendations from the American Diabetes Association (ADA), the European Association for the Study of Diabetes (EASD), the European Society of Cardiology (ESC), and the Kidney Disease: Improving Global Outcomes (KDIGO) initiative advocate preferential use of these therapies according to an individual’s cardiovascular and renal risk profile rather than glycated hemoglobin alone. Lipid-lowering therapy, particularly statins, also remains a cornerstone of cardiovascular prevention in patients with T2DM because dyslipidemia substantially contributes to residual atherosclerotic risk [4,5].

Despite the availability of evidence-based therapies, implementation in routine clinical practice remains inconsistent. Real-world studies from Europe, North America, and Asia have consistently demonstrated lower-than-expected prescription rates of SGLT2 inhibitors and GLP-1 receptor agonists among patients with established cardiovascular disease, chronic kidney disease, or heart failure. Similarly, lipid-lowering therapy is frequently underutilized or insufficiently intensified, resulting in persistent elevations of low-density lipoprotein cholesterol despite clear treatment indications. These evidence-to-practice gaps may reflect multiple factors, including reimbursement policies, treatment cost, prescribing habits, contraindications, adverse effects, patient preference, therapeutic inertia, and limited healthcare resources [6].

Importantly, the presence of guideline-directed pharmacological therapy does not necessarily imply adequate control of the underlying cardio-renal–metabolic abnormalities. Many patients continue to exhibit persistent hyperglycemia, obesity, insulin resistance, dyslipidemia, albuminuria, hypertension, and declining renal function despite receiving contemporary pharmacological treatment. This persistent burden has been described as residual cardio-metabolic or cardio-renal risk and represents one of the major remaining challenges in diabetes management. Residual risk reflects the combined influence of disease duration, progressive β-cell dysfunction, obesity, chronic inflammation, lifestyle factors, medication adherence, treatment intensity, and irreversible vascular damage, emphasizing that pharmacological therapy alone cannot completely eliminate long-term risk [7,8].

Recent attention has therefore shifted toward integrated evaluation of residual risk rather than assessment of individual biochemical targets. Simultaneous consideration of glycemic control, obesity, lipid abnormalities, renal dysfunction, blood pressure, and cardiovascular disease provides a more comprehensive representation of the patient’s clinical status and may identify opportunities for treatment optimization beyond conventional glucose-centered strategies. However, relatively few real-world studies have examined pharmacological treatment patterns together with cumulative residual cardio-renal–metabolic risk within the same patient population [9].

Romania, similar to many Central and Eastern European countries, has experienced a rapid increase in T2DM prevalence together with a substantial burden of obesity, hypertension, cardiovascular disease, and chronic kidney disease. Nevertheless, information regarding the implementation of contemporary glucose- and lipid-lowering therapies and their relationship with persistent residual risk in routine outpatient practice remains limited. Real-world evidence is essential for understanding how international therapeutic recommendations are translated into daily clinical care and for identifying areas where further optimization may be possible [10,11].

Therefore, the present study aimed to characterize real-world pharmacological treatment patterns in adults with T2DM receiving outpatient care and to evaluate the burden of persistent cardio-renal–metabolic risk despite ongoing therapy. Specifically, we sought to (i) describe the use of contemporary glucose- and lipid-lowering therapies, (ii) compare treatment patterns according to major cardio-renal–metabolic phenotypes, (iii) assess residual metabolic and renal abnormalities across treatment groups, (iv) identify independent predictors of persistent residual risk using multivariable regression analyses, and (v) evaluate cumulative residual cardio-renal–metabolic risk together with potential therapeutic gaps in high-risk patient subgroups. We hypothesized that substantial residual cardio-renal–metabolic risk would remain despite widespread pharmacological treatment and that important opportunities for optimization of evidence-based therapy would be identifiable in routine clinical practice.

## 2. Materials and Methods

### 2.1. Study Design and Population

This cross-sectional observational study used routinely collected electronic medical records of adults with type 2 diabetes mellitus (T2DM) attending Oradea County Clinical Emergency Hospital, Romania. The clinical database was prospectively maintained during routine care and retrospectively analyzed after anonymization. We included one eligible outpatient record per adult with T2DM, identified consecutively from visits between 1 October 2024 and 19 February 2026. Because the exported dataset did not retain a visit-level screening log, the index visit was defined for this analysis as the earliest eligible outpatient visit available for each participant in the exported dataset. Data extraction and retrospective analysis were performed between 20 February and 10 March 2026. Each participant contributed one index assessment.

Participants were eligible if they were aged ≥18 years, had confirmed T2DM, and had the essential clinical and treatment information required for the study. Patients with type 1 diabetes, gestational diabetes, or secondary diabetes were excluded, together with records lacking essential eligibility or exposure information. The final analytical cohort comprised 232 unique adults with T2DM and one index visit per participant. Because a contemporaneous screening log was not retained, the total number of records assessed and the numbers excluded for each reason could not be independently verified; accordingly, no reconstructed flow counts are reported. Outcome-specific missing or unusable measurements were handled as described in Section 2.7.

The sample size was determined by the number of consecutive eligible records available during the study period. No a priori sample-size calculation was performed. Accordingly, this was an exploratory analysis of the available clinical cohort, and statistical precision should be interpreted in relation to the sample size and the number of events for each outcome.

Medication exposure was determined from the active prescription list at the index visit. Laboratory values and prescription information were obtained from the linked clinical records. The exact interval between laboratory sampling and prescription documentation could not be reconstructed, limiting assessment of their temporal alignment. The study was reported according to the Strengthening the Reporting of Observational Studies in Epidemiology (STROBE) recommendations for cross-sectional studies.

The lack of a contemporaneous screening log limits assessment of selection bias and is acknowledged as a limitation of the study.

Cohort overlap with a prior publication was assessed explicitly. An earlier report from the same research group used the same 232-participant outpatient cohort and overlapping baseline variables to characterize integrated cardio-renal–metabolic risk and to evaluate machine-learning models for established ASCVD [12]. The present manuscript is a separate secondary analysis of that cohort, focused on pharmacological treatment patterns, treatment-specific residual risk, multivariable correlates of persistent abnormalities, and potential therapeutic gaps; it does not represent a newly recruited cohort.

### 2.2. Clinical Assessment

Demographic variables included age, sex, place of residence, educational level, smoking status, duration of diabetes, and family history of diabetes and cardiovascular disease. Anthropometric evaluation comprised body weight, height, body mass index (BMI), and waist circumference. Blood pressure was measured during the outpatient visit according to routine clinical practice, and systolic and diastolic values were recorded. Clinical comorbidities were ascertained from physician-documented diagnoses, previous specialist reports, hospital discharge summaries, and the active problem list in the electronic medical record. No separate patient questionnaire was used for comorbidity ascertainment; therefore, questionnaire validation was not applicable. No additional independent physician adjudication was performed for this secondary analysis, and comorbidity classification relied on diagnoses documented during routine clinical care.

Clinical comorbidities included hypertension, dyslipidemia, obesity, coronary artery disease, previous myocardial infarction, coronary revascularization, stroke, peripheral arterial disease, heart failure, atrial fibrillation, chronic kidney disease, hepatic steatosis, thyroid disease, chronic pulmonary disease, psychiatric disorders, and malignant diseases. Established atherosclerotic cardiovascular disease (ASCVD) was defined as a documented history of myocardial infarction, coronary revascularization, ischemic stroke, or peripheral arterial disease.

### 2.3. Laboratory Assessment and Definition of Cardio-Renal–Metabolic Phenotypes

Routine biochemical analyses included fasting plasma glucose, glycated hemoglobin (HbA1c), lipid profile, serum creatinine, serum urea, liver enzymes, C-reactive protein, complete blood count, urinary albumin-to-creatinine ratio (UACR), and additional laboratory parameters routinely assessed during diabetes follow-up. Venous blood and spot urine samples were collected as part of routine outpatient diabetes care and analyzed by the clinical laboratory. The present study used the resulting laboratory measurements recorded in the electronic medical records; no additional blood or urine samples were collected specifically for this secondary analysis. Laboratory values were linked to the index prescription record. Renal function was evaluated using the estimated glomerular filtration rate (eGFR), calculated and reported in mL/min/1.73 m^2^. A laboratory-defined chronic kidney disease (CKD) phenotype was defined as an eGFR below 60 mL/min/1.73 m^2^ and/or UACR ≥ 30 mg/g. Because chronicity was not confirmed by repeated measurements for every participant, this manuscript consistently refers to a laboratory-defined CKD phenotype rather than confirmed CKD. Insulin resistance was estimated using the triglyceride–glucose (TyG) index.

SCORE2-Diabetes values were available in the dataset for all 232 participants. However, the model is intended for individuals with T2DM aged 40–69 years without established ASCVD or severe target-organ damage. As the present cohort included participants outside this target population, whole-cohort SCORE2-Diabetes summaries and between-group comparisons were considered exploratory descriptions of recorded scores rather than validated estimates or comparisons of absolute 10-year cardiovascular risk.

For analyses of cumulative residual risk, seven clinically relevant abnormalities were evaluated: HbA1c ≥ 7%, TyG index > 9.04, obesity (BMI ≥ 30 kg/m^2^), blood pressure ≥ 140/90 mmHg, LDL cholesterol ≥ 100 mg/dL, albuminuria (UACR ≥ 30 mg/g), and reduced renal function (eGFR < 60 mL/min/1.73 m^2^).

### 2.4. Pharmacological Treatment Assessment

Current pharmacological therapy was extracted directly from the electronic medical records. Glucose-lowering medications were classified into biguanides, sodium–glucose cotransporter-2 (SGLT2) inhibitors, glucagon-like peptide-1 receptor agonists (GLP-1 RAs), dipeptidyl peptidase-4 (DPP-4) inhibitors, sulfonylureas, insulin regimens, and other antidiabetic medications. Individual SGLT2 inhibitors included dapagliflozin, empagliflozin, and ertugliflozin. Insulin treatment was categorized as basal, rapid-acting, premixed, or combined oral antidiabetic therapy with insulin.

Lipid-lowering therapy was classified into statins, ezetimibe, fibrates, fixed statin–ezetimibe combinations, and proprotein convertase subtilisin/kexin type 9 (PCSK9)-targeted therapies. Because participants frequently received more than one medication, treatment categories were considered non-mutually exclusive.

Therapeutic patterns were subsequently evaluated according to the presence of established ASCVD, heart failure, CKD phenotype, obesity, and a combined high-risk phenotype defined by the presence of at least one of these three major cardio-renal conditions. The database contained separate fields for ACE inhibitors, angiotensin-receptor blockers, beta-blockers, diuretics, and other cardiovascular medications. Mineralocorticoid-receptor antagonists were not separately identifiable in the available structured treatment fields. The present analyses focused on selected glucose- and lipid-lowering therapies and did not evaluate the full spectrum of cardiovascular or renal pharmacotherapy.

### 2.5. Assessment of Residual Cardio-Renal–Metabolic Risk

Residual cardio-metabolic risk was evaluated by comparing metabolic, renal, and cardiovascular parameters according to the principal pharmacological treatment classes. Persistent hyperglycemia was defined as HbA1c ≥ 7%, residual insulin resistance as TyG index > 9.04, residual dyslipidemia as LDL cholesterol ≥ 100 mg/dL, albuminuria as UACR ≥ 30 mg/g, and impaired renal function as eGFR < 60 mL/min/1.73 m^2^.

To summarize the co-occurrence of uncontrolled cardiometabolic and renal abnormalities, an exploratory count was calculated by assigning one point to each of the seven specified abnormalities, yielding a possible range of 0–7. Equal weighting was used to provide a transparent descriptive summary rather than to estimate future event risk. For presentation, participants were grouped into three categories: 0–2, 3–4, and 5–7 abnormalities, representing lower, intermediate, and higher observed abnormality burdens, respectively. These author-defined categories were used to summarize the distribution and compare treatment patterns. The thresholds were not derived from outcome prediction, and no external validation is available for these specific cutoffs. Accordingly, the categories should not be interpreted as validated clinical risk strata or used to guide treatment decisions. HbA1c and TyG, as well as albuminuria and eGFR, capture related biological processes; therefore, the count does not represent seven independent risk domains.

Potential therapeutic gaps were evaluated descriptively by comparing recorded pharmacological treatment with the presence of established ASCVD, heart failure, CKD phenotype, obesity, albuminuria, and elevated LDL cholesterol. These analyses were intended to identify opportunities for individualized therapeutic review rather than assess treatment appropriateness.

### 2.6. Statistical Analysis

Continuous variables were evaluated for normality using the Shapiro–Wilk test together with graphical inspection of histograms and quantile–quantile plots. Normally distributed variables are presented as mean ± standard deviation, whereas skewed variables are reported as median and interquartile range. Categorical variables are presented as frequencies and percentages.

Comparisons between treatment groups were performed using the independent-samples *t*-test or Mann–Whitney U test for continuous variables, depending on data distribution. Comparisons among residual-risk categories were performed using one-way analysis of variance (ANOVA) or the Kruskal–Wallis test as appropriate. Categorical variables were compared using Pearson’s chi-square test or Fisher’s exact test.

Multivariable logistic regression analyses were performed to identify independent correlates of persistent hyperglycemia, elevated TyG index, albuminuria, reduced renal function, and residual hypercholesterolemia. Candidate predictors included age, sex, diabetes duration, BMI/obesity, systolic blood pressure, established ASCVD, and major pharmacological treatment classes. Binary variables were coded as 0 = absent and 1 = present; continuous variables were retained on their original scale, with odds ratios reported per one-year, one-kg/m^2^, or one-mmHg increase as applicable. Variables were selected a priori based on clinical relevance and retained after assessment of multicollinearity. The available-case outcome counts in the supplied CSV were: HbA1c ≥ 7%, N = 231 with 119 events; TyG index > 9.04, N = 232 with 137 events; UACR ≥ 30 mg/g, N = 232 with 80 events; eGFR < 60 mL/min/1.73 m^2^, N = 232 with 59 events; and LDL cholesterol ≥ 100 mg/dL, N = 232 with 127 events. For each model, the analytical sample size, number of outcome events, variables retained in the final model, and reference categories are reported in the expanded Table S1. These values refer to the participants actually included in each fitted model.

Table S1 summarizes the analyzed variable domains, their operational definitions, the statistical methods used for the corresponding comparisons, and, for each regression outcome, the analytical sample, number of events, variables retained in the final model, and reference groups.

### 2.7. Data Quality

One HbA1c value of 114% was present in the supplied CSV and was identified as biologically implausible. The original laboratory report was not provided with the database export; it must be checked for a unit or transcription error before the value is permanently classified as missing. Pending that verification, the value was treated as missing in all analyses involving glycated hemoglobin. After exclusion of this value, 231 HbA1c observations remained. No additional clinically implausible laboratory values requiring exclusion were identified. Missing data were handled using complete-case analysis for each individual statistical model.

### 2.8. Ethical Considerations

The study was conducted in accordance with the Declaration of Helsinki and approved by the Ethical Committee of the Faculty of Medicine and Pharmacy, University of Oradea, Romania (approval No. 42, 31 October 2025). Written informed consent covering the use of clinical data for research was obtained from all participants. Clinical records were anonymized before statistical analysis, and the analytical dataset contained no directly identifying information.

## 3. Results

### 3.1. Real-World Pharmacological Patterns in the Study Population

The study included 232 adults with T2DM receiving routine outpatient care in Romania. The demographic and clinical characteristics of the cohort, together with variable-specific data availability, are summarized in Table S2.

Biguanides represented the most frequently prescribed glucose-lowering therapy and were used by 123 participants (53.0%). Sodium–glucose cotransporter-2 (SGLT2) inhibitors were prescribed to 104 participants (44.8%), including dapagliflozin in 64 (27.6%), empagliflozin in 39 (16.8%), and ertugliflozin in one participant (0.4%). Glucagon-like peptide-1 receptor agonists (GLP-1 RAs) were used by 36 participants (15.5%), whereas dipeptidyl peptidase-4 (DPP-4) inhibitors were prescribed to 34 participants (14.7%). Sulfonylureas were used infrequently (19 participants, 8.2%), while no participants received glinides or thiazolidinediones.

Any insulin therapy was recorded in 75 participants (32.3% of the total cohort). Basal insulin was recorded in 73 participants (31.5%), rapid-acting insulin in 22 (9.5%), and premixed insulin in one (0.4%). These categories were not mutually exclusive, because participants could receive more than one insulin type; consequently, their counts should not be summed to estimate the number of insulin-treated participants.

Fixed-combination treatments were recorded in 78 participants. The most frequent combinations were metformin plus dapagliflozin (34 participants, 14.7%), metformin plus empagliflozin (20, 8.6%), and metformin plus vildagliptin (11, 4.7%). Other combinations included saxagliptin plus dapagliflozin (6, 2.6%), metformin plus sitagliptin (2, 0.9%), and fixed insulin–GLP-1 receptor agonist combinations (6, 2.6%). Percentages refer to the total cohort of 232 participants. Combination categories were not mutually exclusive.

Lipid-lowering therapy also demonstrated marked variability. Statins were prescribed to 113 participants (48.7%), including atorvastatin in 86 participants (37.1%), rosuvastatin in 26 (11.2%), and simvastatin in one participant (0.4%). Ezetimibe was used by 28 participants (12.1%), fibrates by 16 (6.9%), and fixed statin–ezetimibe combinations by 28 participants (12.1%). Proprotein convertase subtilisin/kexin type 9 (PCSK9)-targeted therapies were rarely prescribed, with alirocumab administered to three participants (1.3%), evolocumab to two (0.9%), and inclisiran to one participant (0.4%) (Table 1).

### 3.2. Pharmacological Treatment According to Cardio-Renal–Metabolic Phenotypes

Established ASCVD, heart failure, and the laboratory-defined CKD phenotype were present in 21.1%, 26.3%, and 50.9% of participants, respectively. At least one of these conditions was present in 156 participants (67.2%).

SGLT2 inhibitor use did not differ significantly according to ASCVD, heart failure, or CKD phenotype. GLP-1 receptor agonist use was more frequent among participants with obesity (22.0% vs. 7.6%; *p* = 0.003) and less frequent among those with the CKD phenotype (10.2% vs. 21.1%; *p* = 0.022). Insulin use was more frequent in participants with each of the three cardiovascular or renal phenotypes, while statin-containing therapy was more frequent in those with ASCVD or heart failure. Detailed comparisons are presented in Table 2.

### 3.3. Residual Cardio-Renal–Metabolic Risk According to Pharmacological Treatment

Treatment-specific residual abnormalities are summarized in Table 3. Among SGLT2 inhibitor users, 59/103 participants (57.3%) had HbA1c ≥ 7%, and 29/104 (27.9%) had albuminuria. Median UACR was lower in users than in non-users (14.09 vs. 17.25 mg/g; *p* = 0.010). GLP-1 receptor agonist users had higher BMI and eGFR than non-users.

HbA1c ≥ 7% was more frequent among insulin users: HbA1c ≥ 7% occurred in 63/75 insulin users (84.0%) versus 56/156 non-users (35.9%; *p* < 0.001). Reduced eGFR and albuminuria were also more frequent among insulin users. Although statin-containing therapy was associated with lower LDL cholesterol and TyG values, 38/113 treated participants (33.6%) still had LDL cholesterol ≥ 100 mg/dL (Table 3).

### 3.4. Independent Predictors of Residual Cardio-Metabolic Risk

The multivariable associations are presented in Table 4, with model specifications and reference categories in Table S1. Insulin therapy was associated with higher odds of HbA1c ≥ 7% (aOR 7.41, 95% CI 3.50–15.65; *p* < 0.001), whereas diabetes duration and BMI were not statistically significant in this model. Absence of statin-containing therapy was associated with a TyG index > 9.04 (aOR 2.33, 95% CI 1.36–3.98; *p* = 0.002), while obesity was not.

Insulin therapy was associated with higher odds of albuminuria (aOR 2.23, 95% CI 1.15–4.33; *p* = 0.018), and SGLT2 inhibitor therapy with lower odds (aOR 0.54, 95% CI 0.30–0.95; *p* = 0.033). Diabetes duration and reduced eGFR were not statistically significant in this model. Older age was associated with reduced eGFR (*p* = 0.045), whereas diabetes duration and albuminuria were not. In the LDL cholesterol model, which included all 232 participants, absence of statin-containing therapy was associated with LDL cholesterol ≥ 100 mg/dL (aOR 6.01, 95% CI 3.38–10.66; *p* < 0.001); obesity was not statistically significant.

The multivariable associations are summarized in Table 4, with the complete final adjustment sets and reference categories provided in Table S1. In the HbA1c model, insulin therapy, longer diabetes duration, and higher BMI were associated with HbA1c ≥ 7%. In the TyG model, obesity and absence of statin-containing therapy were associated with a TyG index > 9.04. Albuminuria was associated with longer diabetes duration, insulin therapy, reduced eGFR, and absence of SGLT2 inhibitor therapy. Older age, longer diabetes duration, and albuminuria were associated with reduced eGFR.

The LDL cholesterol model included the full cohort with available LDL measurements rather than only participants receiving lipid-lowering therapy. Absence of statin-containing therapy was associated with LDL cholesterol ≥ 100 mg/dL (aOR 4.21, 95% CI 2.34–7.58), as was obesity (aOR 1.72, 95% CI 1.01–2.94).

### 3.5. Cumulative Residual Cardio-Renal–Metabolic Risk and Therapeutic Gaps

When the seven abnormalities were considered jointly, 167/232 participants (72.4%) had at least three abnormalities and 56/232 (24.1%) had five or more. The lower-, intermediate-, and higher-burden categories included 64, 112, and 56 participants, respectively. Insulin use increased across these categories (21.9%, 31.5%, and 46.4%; *p* = 0.016), whereas the number of major treatment classes did not differ significantly (*p* = 0.924; Table 5).

**Figure 1:**
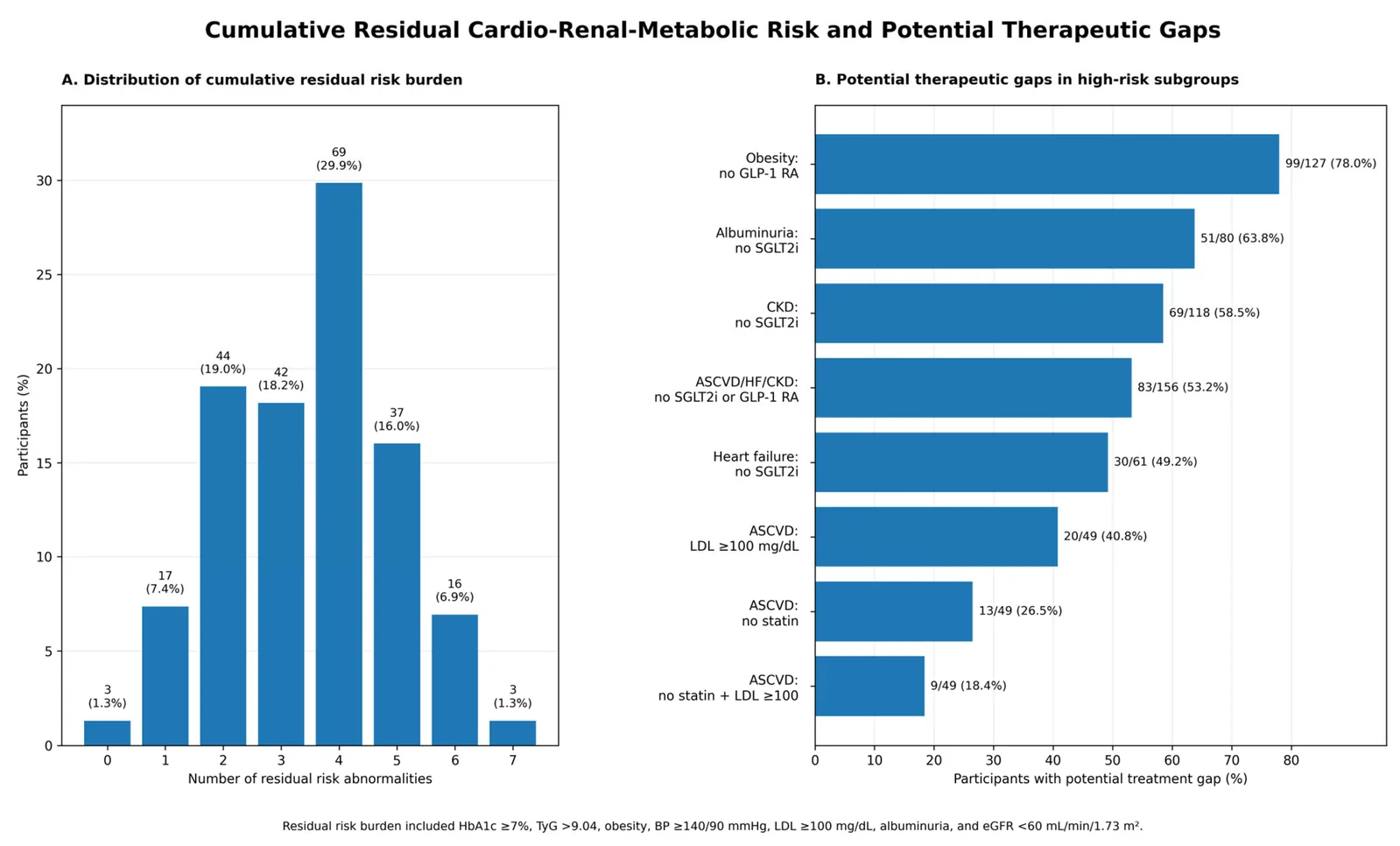
Cumulative residual cardio-renal–metabolic risk and potential therapeutic gaps in adults with type 2 diabetes. Panel (**A**) shows the distribution of the cumulative residual risk burden, defined as the number of persistent abnormalities among HbA1c ≥ 7%, TyG index > 9.04, obesity, blood pressure ≥ 140/90 mmHg, LDL cholesterol ≥ 100 mg/dL, albuminuria ≥ 30 mg/g, and estimated glomerular filtration rate < 60 mL/min/1.73 m^2^. Values above the bars represent absolute frequencies and percentages. Panel (**B**) presents the proportions of participants with potential therapeutic gaps within clinically relevant high-risk subgroups. These descriptive gaps were defined using recorded diagnoses, laboratory abnormalities, and treatment exposure at the time of assessment. They do not account for contraindications, intolerance, affordability, treatment duration, adherence, or patient preference and should not be interpreted as confirmed inappropriate prescribing. The residual-risk analysis included 231 participants after one clinically implausible HbA1c value was treated as missing.

Among participants with ASCVD, heart failure, or the laboratory-defined CKD phenotype, 83/156 (53.2%) received neither an SGLT2 inhibitor nor a GLP-1 receptor agonist. No SGLT2 inhibitor was recorded in 51/80 participants with albuminuria (63.8%), and no statin-containing therapy was recorded in 13/49 participants with ASCVD (26.5%). Potential therapeutic gaps are detailed in Table 6; the abnormality distribution and selected treatment gaps are illustrated in Figure 1.

## 4. Discussion

The present study provides a real-world evaluation of pharmacological treatment patterns and persistent cardio-renal–metabolic risk among adults with T2DM in Romania. This analysis used the same cohort as our earlier report [12], which focused on integrated risk profiling and machine-learning classification of established ASCVD. The present analysis addresses a different clinical question by quantifying treatment use, treatment-specific residual abnormalities, independent correlates of persistent risk, and potential therapeutic gaps. Although contemporary glucose-lowering and lipid-lowering therapies were widely prescribed, clinically important residual metabolic and renal abnormalities remained common across treatment groups (Table 1, Table 2 and Table 3). These findings highlight that treatment exposure alone does not necessarily translate into achievement of therapeutic targets and support continuous risk reassessment beyond prescription patterns.

### 4.1. Contemporary Pharmacological Management in Routine Clinical Practice

Biguanides remained the most frequently prescribed glucose-lowering therapy, while SGLT2 inhibitors represented the second most common treatment class (Table 1). Approximately 45% of participants received an SGLT2 inhibitor, whereas only 15.5% were treated with a GLP-1 receptor agonist. These prescribing patterns describe implementation in routine outpatient care and should not be interpreted as measures of treatment efficacy [13,14,15,16].

Nevertheless, important opportunities for optimization remain. Despite the high prevalence of obesity, chronic kidney disease phenotype, heart failure, and established atherosclerotic cardiovascular disease within the cohort, many patients with these conditions were not receiving therapies currently recommended by international guidelines (Table 2 and Table 6).

The relatively low utilization of GLP-1 receptor agonists likely reflects several real-world barriers, including reimbursement policies, treatment cost, injectable administration, patient acceptance, and prescribing restrictions rather than lack of clinical indication alone. Consequently, treatment availability and healthcare-system factors should be considered when interpreting these findings [17,18,19].

### 4.2. Residual Cardio-Metabolic Risk Persists Despite Pharmacological Treatment

One of the principal findings of this study is the persistence of substantial residual cardio-metabolic risk despite pharmacological treatment (Table 3). More than half of SGLT2 inhibitor users continued to exhibit HbA1c values ≥ 7%, almost one-third remained albuminuric, and clinically important proportions of statin-treated participants still had LDL cholesterol concentrations above the descriptive threshold used in this study [20].

These observations should not be interpreted as treatment failure. Instead, they likely reflect the complexity of T2DM management, in which disease duration, progressive β-cell dysfunction, obesity, medication adherence, lifestyle factors, therapeutic inertia, dose intensity, and comorbidity burden collectively influence treatment outcomes. Patients receiving more intensive therapies frequently represent those with more advanced disease and inherently higher baseline risk, a phenomenon commonly referred to as confounding by indication [21,22,23].

Compared with participants without recorded insulin therapy, insulin-treated participants more frequently had HbA1c ≥ 7%, eGFR < 60 mL/min/1.73 m^2^, and albuminuria (Table 3). These cross-sectional differences may reflect confounding by indication and greater disease severity among patients requiring insulin. This finding is consistent with clinical practice, where insulin therapy is generally introduced in patients with longer disease duration, greater metabolic deterioration, or failure of previous therapeutic strategies rather than causing these adverse characteristics [24,25,26].

### 4.3. Independent Predictors of Persistent Risk

Multivariable analyses identified associations between selected treatment exposures and prevalent cardiometabolic abnormalities. Insulin therapy was associated with higher odds of hyperglycemia and albuminuria, while SGLT2 inhibitor therapy was associated with lower odds of albuminuria. Absence of statin-containing therapy was associated with higher odds of elevated LDL cholesterol and TyG index. These findings may reflect differences in disease severity, treatment selection, and other unmeasured factors and should not be interpreted as causal treatment effects [27,28,29].

Older age was associated with reduced eGFR. Diabetes duration, BMI or obesity, and the reciprocal associations between albuminuria and reduced eGFR were not statistically significant in their respective models. The inverse association between SGLT2 inhibitor use and albuminuria is compatible with evidence from randomized trials, but the present cross-sectional analysis cannot establish a treatment benefit.

### 4.4. Cumulative Residual Risk and Therapeutic Opportunities

An important strength of the present study is the evaluation of cumulative residual cardio-renal–metabolic risk rather than isolated clinical outcomes (Table 5 and Figure 1). The count is exploratory, equally weighted, and not externally validated; it should not be described as a validated risk score or as a predictor of future cardiovascular or renal events. Nearly three-quarters of participants exhibited at least three persistent abnormalities, while approximately one-quarter presented five or more concurrent abnormalities.

The analysis also identified several potential therapeutic gaps (Table 6 and Figure 1). More than half of participants with established ASCVD, heart failure, or chronic kidney disease were not receiving either an SGLT2 inhibitor or a GLP-1 receptor agonist. Similarly, over one-quarter of patients with established ASCVD had no recorded statin-containing therapy, and approximately two-thirds of albuminuric participants were not receiving an SGLT2 inhibitor [30,31,32,33].

These observations should be interpreted cautiously. The present database did not include information regarding contraindications, medication intolerance, reimbursement eligibility, affordability, previous treatment discontinuation, patient preference, adherence, or dose intensity. Consequently, these findings identify potential opportunities for individualized therapeutic review rather than definitive evidence of undertreatment or inappropriate prescribing.

### 4.5. Clinical Implications

The findings reinforce the concept that management of T2DM should extend beyond achievement of isolated glycemic targets. Residual cardiometabolic and renal abnormalities remained common among participants receiving contemporary therapies (Table 3), while multiple concurrent abnormalities were present in a substantial proportion of the cohort (Table 5). These findings support assessing glycemic, lipid, blood-pressure, obesity, and renal parameters together during clinical review [34,35]. SCORE2-Diabetes was summarized across the entire cohort, including participants outside its intended population. This limits the clinical interpretation of the reported scores and their comparisons between treatment groups.

Routine evaluation of cumulative residual risk may facilitate earlier identification of patients requiring treatment optimization, multidisciplinary intervention, or intensified follow-up. Such an integrated approach aligns with contemporary cardio-renal–metabolic care models and may improve implementation of evidence-based therapies in routine clinical practice.

### 4.6. Strengths and Limitations

This study has several strengths. It was based on a well-characterized real-world cohort and evaluated both pharmacological treatment patterns and residual cardio-renal–metabolic risk using standardized clinical and laboratory data. The analyses combined descriptive epidemiology, subgroup comparisons, multivariable regression, and assessment of cumulative residual risk, providing a comprehensive overview of current diabetes management in routine practice.

Several limitations should also be acknowledged. First, the cross-sectional design precludes causal inference and does not permit evaluation of temporal relationships between treatment initiation and clinical outcomes. Second, pharmacological exposure was assessed at a single time point, without information regarding treatment duration, medication adherence, dose escalation, previous therapies, or discontinuation. Third, contraindications, intolerance, reimbursement restrictions, socioeconomic status, and patient preference were unavailable and may explain some apparent therapeutic gaps. Fourth, the study was conducted in a single outpatient population, which may limit generalizability to other healthcare systems or clinical settings. Finally, although multivariable adjustment was performed, residual confounding cannot be completely excluded.

The absence of a contemporaneous screening log prevented independent verification of the total number of records screened and the reasons for exclusion, limiting assessment of potential selection bias.

Future prospective multicenter studies should evaluate longitudinal changes in residual cardio-metabolic risk following optimization of pharmacological therapy and determine whether integrated assessment of treatment gaps improves cardiovascular and renal outcomes.

## 5. Conclusions

In this real-world cohort of adults with type 2 diabetes mellitus, contemporary glucose- and lipid-lowering therapies were widely used; however, clinically important residual cardio-renal–metabolic risk remained common. Persistent hyperglycemia, obesity, dyslipidemia, albuminuria, and impaired renal function frequently coexisted despite ongoing pharmacological treatment.

Insulin therapy was associated with higher odds of hyperglycemia and albuminuria, while SGLT2 inhibitor therapy was associated with lower odds of albuminuria. Absence of statin-containing therapy was associated with higher odds of elevated LDL cholesterol and TyG index, and older age was associated with reduced eGFR. These cross-sectional associations do not establish causal treatment effects.

The study also identified potential opportunities for treatment optimization, particularly among patients with established cardiovascular or renal disease who were not receiving therapies with proven cardio-renal benefit. These observations should be interpreted within the context of real-world clinical practice and not as evidence of inappropriate prescribing, since treatment eligibility, contraindications, adherence, affordability, and therapeutic history were unavailable.

Overall, the findings emphasize that prescription of evidence-based therapies alone is insufficient to eliminate residual cardio-metabolic risk. Comprehensive, individualized, and repeatedly reassessed cardio-renal–metabolic management remains essential for improving long-term outcomes in adults with type 2 diabetes mellitus. Prospective multicenter studies are warranted to validate these observations and determine whether systematic identification of residual risk and therapeutic gaps translates into better clinical outcomes.

## Figures and Tables

**Table 1:** Pharmacological Treatment Patterns in the Study Population (N = 232).

Pharmacological Therapy	*n* (%)
Glucose-lowering therapy	
Biguanides	123 (53.0)
SGLT2 inhibitors (overall)	104 (44.8)
• Dapagliflozin	64 (27.6)
• Empagliflozin	39 (16.8)
• Ertugliflozin	1 (0.4)
GLP-1 receptor agonists	36 (15.5)
DPP-4 inhibitors	34 (14.7)
Sulfonylureas	19 (8.2)
Glinides	0 (0.0)
Thiazolidinediones	0 (0.0)
Basal insulin	73 (31.5)
Rapid-acting insulin	22 (9.5)
Premixed insulin	1 (0.4)
Diet only	27 (11.6)
Most frequent fixed combinations	
Metformin + dapagliflozin	34 (14.7)
Metformin + empagliflozin	20 (8.6)
Metformin + vildagliptin	6 (2.6)
Saxagliptin + dapagliflozin	6 (2.6)
Lipid-lowering therapy	
Any statin	113 (48.7)
• Atorvastatin	86 (37.1)
• Rosuvastatin	26 (11.2)
• Simvastatin	1 (0.4)
Ezetimibe	28 (12.1)
Fibrates	16 (6.9)
Fixed statin–ezetimibe combination	28 (12.1)
Alirocumab	3 (1.3)
Evolocumab	2 (0.9)
Inclisiran	1 (0.4)

Percentages are calculated using the total study population (N = 232). Participants could receive more than one pharmacological agent; therefore, percentages do not sum to 100%.

**Table 2:** Pharmacological Treatment Patterns by Cardiovascular, Renal, and Obesity Status in Patients with Type 2 Diabetes.

Clinical Phenotype	SGLT2 Inhibitor, n/N (%)	GLP-1 Receptor Agonist, n/N (%)	SGLT2 Inhibitor or GLP-1 Receptor Agonist, n/N (%)	Any Insulin, n/N (%)	Statin-Containing Therapy, n/N (%)
Established ASCVD present	25/49 (51.0)	5/49 (10.2)	26/49 (53.1)	27/49 (55.1)	36/49 (73.5)
Established ASCVD absent	79/183 (43.2)	31/183 (16.9)	92/183 (50.3)	48/183 (26.2)	77/183 (42.1)
*p* value	0.326	0.247	0.729	<0.001	<0.001
Heart failure present	31/61 (50.8)	7/61 (11.5)	33/61 (54.1)	28/61 (45.9)	40/61 (65.6)
Heart failure absent	73/171 (42.7)	29/171 (17.0)	85/171 (49.7)	47/171 (27.5)	73/171 (42.7)
*p* value	0.273	0.310	0.556	0.008	0.002
CKD phenotype present	49/118 (41.5)	12/118 (10.2)	52/118 (44.1)	47/118 (39.8)	63/118 (53.4)
CKD phenotype absent	55/114 (48.2)	24/114 (21.1)	66/114 (57.9)	28/114 (24.6)	50/114 (43.9)
*p* value	0.303	0.022	0.035	0.013	0.147
Obesity present	64/127 (50.4)	28/127 (22.0)	75/127 (59.1)	34/127 (26.8)	60/127 (47.2)
Obesity absent	40/105 (38.1)	8/105 (7.6)	43/105 (41.0)	41/105 (39.0)	53/105 (50.5)
*p* value	0.061	0.003	0.006	0.047	0.624
Any ASCVD, heart failure, or CKD phenotype	67/156 (42.9)	20/156 (12.8)	73/156 (46.8)	59/156 (37.8)	86/156 (55.1)
None of these phenotypes	37/76 (48.7)	16/76 (21.1)	45/76 (59.2)	16/76 (21.1)	27/76 (35.5)
*p* value	0.410	0.104	0.076	0.010	0.005

Data are presented as treated participants/total participants within each phenotype category (percentage). Established ASCVD was defined as previous myocardial infarction, coronary revascularization, stroke, or peripheral arterial disease. The chronic kidney disease phenotype was defined as an estimated glomerular filtration rate < 60 mL/min/1.73 m^2^ and/or urinary albumin-to-creatinine ratio ≥ 30 mg/g. Statin-containing therapy included statin monotherapy and fixed statin–ezetimibe combinations. Comparisons were performed using Pearson’s chi-square test or Fisher’s exact test, as appropriate. Treatment categories were not mutually exclusive. Abbreviations: ASCVD, atherosclerotic cardiovascular disease; CKD, chronic kidney disease; GLP-1, glucagon-like peptide-1; SGLT2, sodium–glucose cotransporter-2.

**Table 3:** Residual cardio-renal–metabolic risk according to major pharmacological therapies.

Parameter	Treatment Users	Treatment Non-Users	*p* Value
SGLT2 inhibitor therapy	n = 104	n = 128	
HbA1c, %	7.30 (6.48–8.50)	6.80 (6.18–8.85)	0.245
HbA1c ≥ 7%	60/104 (57.7%)	60/128 (46.9%)	0.101
TyG index	9.15 (8.79–9.70)	9.14 (8.80–9.72)	0.706
eGFR, mL/min/1.73 m^2^	80.60 (57.34–94.08)	85.17 (63.63–96.43)	0.138
UACR, mg/g	14.09 (10.87–32.72)	17.25 (11.87–78.63)	0.010
Albuminuria ≥ 30 mg/g	29/104 (27.9%)	51/128 (39.8%)	0.057
GLP-1 receptor agonist therapy	n = 36	n = 196	
Body mass index, kg/m^2^	33.85 (30.32–36.70)	30.00 (26.76–33.26)	<0.001
Obesity	28/36 (77.8%)	99/196 (50.5%)	0.003
HbA1c, %	6.65 (6.08–8.73)	7.10 (6.30–8.60)	0.316
eGFR, mL/min/1.73 m^2^	91.03 (80.74–99.25)	80.43 (57.87–94.08)	0.007
SCORE2-Diabetes, %	29.10 (17.13–36.00)	35.70 (23.45–47.10)	0.009
Any insulin therapy	n = 75 (HbA1c available)	n = 157 (156 HbA1c available)	
HbA1c, %	8.50 (7.30–9.70)	6.50 (6.10–7.70)	<0.001
HbA1c ≥ 7%	63/75 (84.0%)	57/157 (36.3%)	<0.001
eGFR, mL/min/1.73 m^2^	69.95 (55.23–91.60)	85.50 (65.00–96.00)	0.008
eGFR < 60 mL/min/1.73 m^2^	26/75 (34.7%)	33/157 (21.0%)	0.026
Albuminuria ≥ 30 mg/g	33/75 (44.0%)	47/157 (29.9%)	0.035
SCORE2-Diabetes, %	41.70 (33.95–51.85)	30.70 (20.50–42.90)	<0.001
Statin-containing therapy	n = 113	n = 119	
LDL cholesterol, mg/dL	90.25 (62.63–109.29)	124.83 (99.81–149.23)	<0.001
LDL cholesterol ≥ 100 mg/dL	38/113 (33.6%)	89/119 (74.8%)	<0.001
TyG index	9.02 (8.67–9.42)	9.36 (8.90–9.93)	<0.001
TyG index > 9.04	55/113 (48.7%)	82/119 (68.9%)	0.002
HbA1c, %	7.10 (6.30–8.60)	6.90 (6.20–8.60)	0.831

Continuous variables are presented as median (interquartile range), and categorical variables as number/available participants (percentage). Comparisons were performed using the Mann–Whitney U test for continuous variables and Pearson’s chi-square or Fisher’s exact test for categorical variables. Treatment groups were not mutually exclusive. One implausible HbA1c value was treated as missing. For HbA1c analyses, the available sample sizes for treatment users/non-users were 103/128 for SGLT2 inhibitors, 36/195 for GLP-1 receptor agonists, 75/156 for insulin, and 113/118 for statin-containing therapy. The analyses are unadjusted and cross-sectional; consequently, differences between treatment users and non-users may reflect confounding by indication, disease severity, contraindications, treatment duration, adherence, and other unmeasured factors. SCORE2-Diabetes comparisons are exploratory and include participants outside the model’s intended population; they should not be interpreted as differences in validated absolute cardiovascular risk. Abbreviations: eGFR, estimated glomerular filtration rate; GLP-1, glucagon-like peptide-1; HbA1c, glycated hemoglobin; LDL, low-density lipoprotein; SGLT2, sodium–glucose cotransporter-2; TyG, triglyceride–glucose; UACR, urinary albumin-to-creatinine ratio. The insulin comparison included 75 users and 157 non-users. All percentages in the table use the available-case denominator for the relevant outcome.

**Table 4:** Multivariable Predictors of Residual Cardio-Metabolic Risk.

Outcome	Independent Predictor	Adjusted OR	95% CI	*p* Value
HbA1c ≥ 7%	Insulin therapy	7.41	3.50–15.65	<0.001
	Diabetes duration (per year)	1.04	0.99–1.09	0.108
	Body mass index (per kg/m^2^)	1.00	0.95–1.06	0.944
TyG index >9.04	Obesity	1.31	0.77–2.25	0.320
	No statin-containing therapy	2.33	1.36–3.98	0.002
Albuminuria ≥30 mg/g	Diabetes duration (per year)	0.99	0.95–1.03	0.513
	Insulin therapy	2.23	1.15–4.33	0.018
	eGFR < 60 mL/min/1.73 m^2^	1.01	0.53–1.92	0.980
	SGLT2 inhibitor therapy	0.54	0.30–0.95	0.033
eGFR < 60 mL/min/1.73 m^2^	Age (per year)	1.03	1.00–1.07	0.045
	Diabetes duration (per year)	1.01	0.97–1.05	0.500
	Albuminuria ≥ 30 mg/g	1.05	0.56–1.96	0.882
LDL cholesterol ≥ 100 mg/dL	No statin-containing therapy	6.01	3.38–10.66	<0.001
	Obesity	0.69	0.39–1.23	0.213

Adjusted odds ratios were estimated using multivariable logistic regression. Candidate predictors included age, sex, diabetes duration, body mass index, systolic blood pressure, SGLT2 inhibitor therapy, GLP-1 receptor agonist therapy, insulin therapy, statin-containing therapy, and clinically relevant comorbidities. Final models retained the variables listed in Table S1 after assessment for multicollinearity; the corresponding reference categories and scaling of continuous predictors are reported there. Because the study was cross-sectional, the reported associations should not be interpreted as causal treatment effects but as factors associated with persistent residual cardio-metabolic risk. Model-specific available-case outcome counts were HbA1c ≥ 7%, N = 231/119 events; TyG index > 9.04, N = 232/137; albuminuria, N = 232/80; eGFR < 60 mL/min/1.73 m^2^, N = 232/59; and LDL cholesterol ≥ 100 mg/dL, N = 232/127. The LDL cholesterol model was fitted in the full cohort with available LDL values, not only among statin-treated participants.

**Table 5:** Cumulative residual cardio-renal–metabolic risk burden and major pharmacological treatment patterns.

Characteristic	Low Burden, 0–2 Abnormalities (n = 64)	Moderate Burden, 3–4 Abnormalities (n = 112)	High Burden, 5–7 Abnormalities (n = 56)	*p* Value
Residual abnormalities, mean ± SD	1.64 ± 0.57	3.63 ± 0.49	5.39 ± 0.59	—
SGLT2 inhibitor	25 (39.1%)	55 (49.1%)	24 (42.9%)	0.449
GLP-1 receptor agonist	13 (20.3%)	15 (13.4%)	8 (14.3%)	0.467
SGLT2 inhibitor or GLP-1 receptor agonist	30 (46.9%)	59 (52.7%)	29 (51.8%)	0.776
Any insulin therapy	14 (21.9%)	35 (31.3%)	26 (46.4%)	0.016
Statin-containing therapy	37 (57.8%)	55 (49.1%)	21 (37.5%)	0.084
Number of major treatment classes, mean	1.39	1.43	1.41	0.924

The residual risk count incorporated HbA1c ≥ 7%, TyG index > 9.04, obesity, blood pressure ≥ 140/90 mmHg, LDL cholesterol ≥ 100 mg/dL, albuminuria ≥ 30 mg/g, and eGFR < 60 mL/min/1.73 m^2^. Major treatment classes included SGLT2 inhibitors, GLP-1 receptor agonists, insulin, and statin-containing therapy. Comparisons of categorical variables were performed using Pearson’s chi-square test, while the number of treatment classes was compared using the Kruskal–Wallis test. One participant was excluded because of an implausible HbA1c value. The residual risk count is exploratory and has not been externally validated.

**Table 6:** Potential Therapeutic Gaps Among Participants With High-Risk Clinical Phenotypes.

High-Risk Subgroup	Potential Therapeutic Gap	n/N (%)
ASCVD, heart failure, or CKD	Neither SGLT2 inhibitor nor GLP-1 receptor agonist	83/156 (53.2%)
CKD phenotype	No SGLT2 inhibitor	69/118 (58.5%)
Albuminuria ≥ 30 mg/g	No SGLT2 inhibitor	51/80 (63.8%)
Heart failure	No SGLT2 inhibitor	30/61 (49.2%)
Established ASCVD	No statin-containing therapy	13/49 (26.5%)
Established ASCVD	LDL cholesterol ≥ 100 mg/dL	20/49 (40.8%)
Established ASCVD	No statin and LDL cholesterol ≥ 100 mg/dL	9/49 (18.4%)
Obesity	No GLP-1 receptor agonist	99/127 (78.0%)

Potential treatment gaps were defined descriptively using recorded diagnoses, laboratory abnormalities, and pharmacological treatment at the time of assessment. They do not account for contraindications, intolerance, patient preference, affordability, treatment initiation date, dose intensity, or adherence and should therefore not be interpreted as confirmed undertreatment. Abbreviations: ASCVD, atherosclerotic cardiovascular disease; CKD, chronic kidney disease; GLP-1, glucagon-like peptide-1; LDL, low-density lipoprotein; SGLT2, sodium–glucose cotransporter-2.

## Data Availability

The original contributions presented in this study are included in the article/Supplementary Materials. Further inquiries can be directed to the corresponding author.

## Supplementary Materials

The following supporting information can be downloaded at: https://www.mdpi.com/article/10.3390/diseases14100387/s1, Table S1. Variables and statistical tests used in the analysis; Table S2. Overall clinical characteristics of the study cohort.

## Abbreviations

The following abbreviations are used in this manuscript:

AUC	Area under the curve
BMI	Body mass index
CI	Confidence interval
CRP	C-reactive protein
DPP-4	Dipeptidyl peptidase-4
eGFR	Estimated glomerular filtration rate
FIB-4	Fibrosis-4 index
GLP-1	Glucagon-like peptide-1
HbA1c	Glycated hemoglobin
HDL	High-density lipoprotein
IQR	Interquartile range
LDL	Low-density lipoprotein
OR	Odds ratio
ROC	Receiver operating characteristic
SD	Standard deviation
SGLT2	Sodium–glucose cotransporter-2
T0	Baseline assessment
T1	Six-month follow-up assessment
T2DM	Type 2 diabetes mellitus
TyG	Triglyceride–glucose index
UACR	Urinary albumin-to-creatinine ratio
