# Supplementary material for: Real-World Pharmacological Patterns and Residual Cardiometabolic Risk in Patients with Type 2 Diabetes in Romania
Source: Diseases. 2026 Oct 21;14(10):387. doi: 10.3390/diseases14100387 (PMC13702964; doi:10.3390/diseases14100387)
Supplement: Supplementary file 1 [file diseases-14-00387-s001.zip › diseases-4520154-supplementary.pdf]

Table S1. Variables and statistical tests used in the analysis.

Variable/domain	Operational definition or summary	Statistical test/model
Continuous varia-bles	Mean ± SD if normally distributed; median (IQR) otherwise	Shapiro–Wilk/QQ plot; t-test or Mann–Whitney U; ANOVA or Kruskal–Wallis
Categorical variables	n (%)	Pearson chi-square or Fisher exact test
Persistent hypergly-cemia	HbA1c >=7%	Multivariable logistic regression
Insulin resistance	TyG index >9.04	Multivariable logistic regression
Albuminuria	UACR >=30 mg/g	Multivariable logistic regression
Reduced renal func-tion	eGFR <60 mL/min/1.73 m²	Multivariable logistic regression
Residual hypercho-lesterolemia	LDL cholesterol >=100 mg/dL	Multivariable logistic regression
Exploratory abnor-mality count	Seven binary abnormalities; equal-weight descriptive count	Descriptive distribution; chi-square/Kruskal–Wallis

Table S2. Overall clinical characteristics of the study cohort.

Variable	Summary	Available N	Missing
Age, years	64.0 (55.0–70.0)	232	0
Female sex	122 (52.6%)	232	0
Diabetes duration, years	6.0 (2.0–14.0)	232	0
Current smoking	29 (12.5%)	232	0
Former smoking	40 (17.2%)	232	0
Never smoking	163 (70.3%)	232	0
BMI, kg/m²	30.40 (27.11–34.13)	232	0
Obesity (BMI >=30 kg/m²)	127 (54.7%)	232	0
Systolic blood pressure, mmHg	145.0 (130.0–159.25)	232	0
Diastolic blood pressure, mmHg	85.0 (79.0–95.0)	232	0
HbA1c, %	7.00 (6.25–8.60)	231	1*
LDL cholesterol, mg/dL	108.46 (83.36–133.81)	232	0
eGFR, mL/min/1.73 m²	82.96 (59.90–94.89)	232	0
UACR, mg/g	15.87 (11.49–46.91)	232	0
Established ASCVD	49 (21.1%)	232	0
Heart failure	61 (26.3%)	232	0
Laboratory-defined CKD phenotype	118 (50.9%)	232	0
